# Head and Neck Radiotherapy and Dentomaxillofacial Diagnostic Imaging: Biological Interactions and Protective Approaches

**DOI:** 10.3390/biomedicines13123046

**Published:** 2025-12-11

**Authors:** Cyro Daniel Hikaro Fuziama, Ana Cristina Borges-Oliveira, Lana Ferreira Santos, Sérgio Lúcio Pereira de Castro Lopes, Andre Luiz Ferreira Costa

**Affiliations:** 1 Postgraduate Program in Dentistry, Dentomaxillofacial Radiology and Imaging Laboratory, Cruzeiro do Sul University (UNICSUL), São Paulo 01506-000, Brazil; drcyrofuziama@gmail.com; 2 Department of Social and Preventive Dentistry, School of Dentistry, Universidade Federal de Minas Gerais, Belo Horizonte 31270-901, Brazil; anacboliveira@ufmg.br; 3 Department of Diagnosis and Surgery, The Institute of Sciences and Technology of São Paulo State University (UNESP), São José dos Campos 12245-000, Brazil; lana.santos@unesp.br (L.F.S.); sergio.lopes@unesp.br (S.L.P.d.C.L.); 4 Department of Anesthesiology, Oncology and Radiology, Faculty of Medical Sciences, University of Campinas (UNICAMP), Campinas 13083-887, Brazil

**Keywords:** head and neck squamous cell carcinoma, oral mucositis, precision oncology, radiation injury, radiobiology

## Abstract

Radiotherapy is a fundamental component in the management of head and neck malignancies, but its non-selective effects on surrounding normal tissues can result in significant oral complications. The oral cavity and oropharynx contain several radiosensitive structures, including mucosa, salivary glands, and alveolar bone, which are susceptible to both acute and late toxicities resulting in mucositis, xerostomia, and osteoradionecrosis. Although dentomaxillofacial diagnostic imaging, such as intraoral radiography, panoramic imaging and cone-beam computed tomography (CBCT), delivers radiation doses several orders of magnitude lower than therapeutic exposures, its biological impact on previously irradiated tissues remains underexplored. Even low-dose X-rays may act as secondary stressors, reactivating oxidative and inflammatory pathways in tissues with compromised repair capacity. In this review, we examine the radiobiological and dosimetric implications of using diagnostic ionizing imaging in patients undergoing or recently having completed head and neck radiotherapy. We summarize current evidence on potential additive effects of low-dose imaging, emphasizing the importance of justification, timing, and protocol optimization. Finally, we discuss radioprotective strategies (e.g., dose modulation, field limitation, and integration of modern low-dose imaging technologies) designed to reduce unnecessary exposure, thus enhancing tissue preservation and ensuring diagnostic safety in this vulnerable patient population

## 1. Introduction

Advances in radiotherapy have significantly improved the survival of patients with head and neck malignancies. Nevertheless, ionizing radiation can induce acute and late toxicities in oral and maxillofacial structures (i.e., mandible, mucosa, salivary glands, and dentition) requiring continuous dentomaxillofacial imaging for diagnosis, planning, and rehabilitation [1,2,3].

In clinical practice, intraoral and panoramic radiographs are commonly used for caries control, periodontal assessment, and endodontic follow-up, whereas cone-beam computed tomography (CBCT) is primarily indicated to evaluate bone integrity, pathological alterations, implant planning, or suspected osteoradionecrosis [4,5,6,7,8]. Despite their diagnostic value, the biological safety of performing additional radiographic examinations in patients undergoing radiotherapy remains underexplored.

This review addresses a neglected intersection among radiobiology, radiotherapy physics and dentomaxillofacial imaging by focusing on (A) the magnitude of additional exposure, (B) radiobiological plausibility of cumulative effects, and (C) clinical and ethical recommendations for safe imaging in oncological patients.

Although radiotherapy and radiology share the same physical principle (i.e., ionizing radiation) for diagnosis, their biological impact and clinical intent differ substantially. Radiotherapy delivers therapeutic doses designed to induce DNA damage and cellular apoptosis within tumor tissues [9,10], whereas radiology (e.g., dentomaxillofacial imaging) operates at a range of milligray, aiming to generate diagnostic information with minimal exposure [11]. However, irradiated regions containing tissues such as bone, mucosa, and salivary glands often exhibit reduced vascularity, cellularity and repair capacity, thus causing them to be potentially more vulnerable even to low-dose exposures [12]. This raises important questions about whether conventional assumptions of safety for diagnostic imaging remain entirely applicable in the context of ongoing or recent radiotherapy.

Furthermore, the increasing use of high-resolution imaging modalities, particularly CBCT and digital panoramic systems, has improved diagnostic precision but has also renewed discussions regarding cumulative radiation exposure, especially in younger or long-term cancer survivors [13,14]. Modern oncological care therefore requires an integrated understanding of how repeated imaging interacts biologically with tissues previously altered by therapeutic radiation [15]. Yet only a few studies have quantified these additive effects or proposed evidence-based imaging protocols specifically tailored for this population. Bridging these knowledge gaps demands a multidisciplinary effort that unites dental radiologists, radiation oncologists, and medical physicists to harmonize diagnostic necessity and radiobiological safety within comprehensive head and neck cancer care [16,17].

Despite current understanding of the biological effects of head and neck radiotheapy, the potential additive impact of low-dose dentomaxillofacial imaging on previously irradiated tissues remains largely unexplored. Existing literature addresses radiation-induced toxicity or dental management in oncology patients, but does not clarify how intraoral radiographs, panoramic imaging, or CBCT interact biologically with tissues already compromised by therapeutic exposure. The novelty of this review lies in examining this overlooked interface by integrating radiobiological mechanisms, dosimetric evidence, and clinical decision-making principles. By defining when low-dose imaging is safe, when caution is warranted, and which protective strategies should be adopted, this review fills an important gap and provides a more biologically informed framework for imaging in irradiated patients.

## 2. Methods

We conducted a narrative literature review using PubMed, Scopus, Web of Science, and Google Scholar, covering publications from 2000 to 2025, which reflects the era of modern radiotherapy and contemporary dentomaxillofacial imaging technologies. Only studies published in English were considered.

Search terms combined anatomical descriptors of the dentomaxillofacial region with concepts related to radiotherapy-induced toxicity, diagnostic imaging dose, cone-beam computed tomography, panoramic radiography, radiobiological effects, radioprotection, and cumulative exposure. The initial search retrieved approximately 500–600 records. After screening titles and abstracts for relevance to diagnostic imaging performed during or after head and neck radiotherapy, around 150–180 articles were selected for full-text evaluation.

Following assessment of eligibility, approximately 70–80 studies were incorporated into the final synthesis, corresponding to the number of works ultimately cited in this review. Publications were excluded if they focused exclusively on non-ionizing imaging, were unrelated to the head and neck region, consisted solely of case reports, or lacked biological or dosimetric relevance to irradiated tissues. Reference lists of key articles were also examined to ensure comprehensive coverage.

This approach enabled the integration of experimental, dosimetric, radiobiological, and clinical evidence, providing a coherent overview of the potential additive effects of diagnostic imaging in irradiated tissues.

## 3. Structure of the Oral and Maxillofacial Region

**Figure 1:**
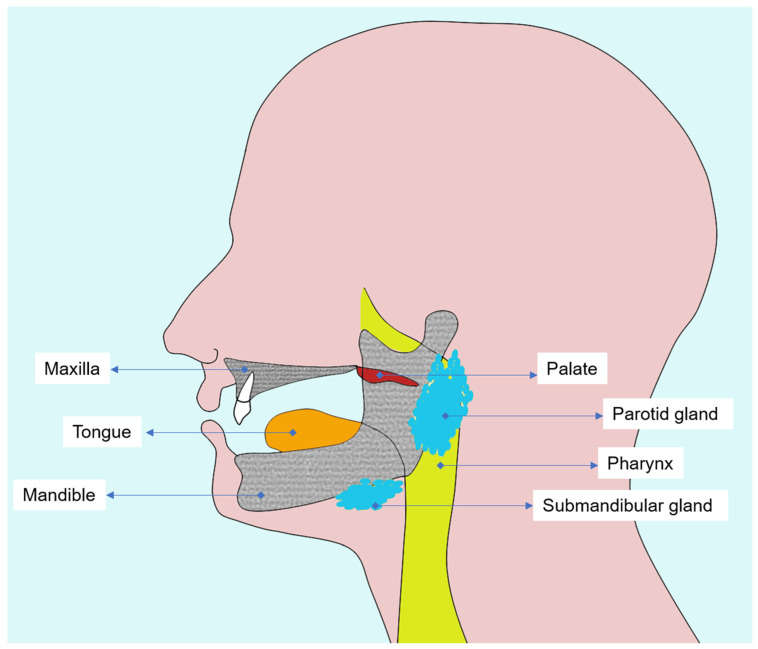
Diagram illustrating the main tissues affected by head and neck radiotherapy, emphasizing oral mucosa, salivary glands, and alveolar and mandibular bone.

A thorough understanding of the oral and maxillofacial anatomy is essential to interpret radiotherapy-induced changes and to plan diagnostic imaging in head and neck cancer patients (Figure 1). Both oral mucosa and alveolar bone represent the most radiosensitive tissues in the irradiated field, in which cellular renewal and vascular dynamics are profoundly altered by ionizing radiation.

The oral mucosa, composed of stratified squamous epithelium and an underlying connective tissue layer (lamina propria), is highly proliferative and rapidly responds to radiation injury [18]. During radiotherapy, direct DNA damage and microvascular inflammation lead to epithelial thinning, erythema, and ulceration [19,20]. These changes culminate in oral mucositis, a frequent acute toxicity which may reach nearly all patients treated for head and neck tumors [21].

Beneath the mucosa, the maxilla and mandible form the osseous framework of the oral cavity and are critical structures for dental and prosthetic rehabilitation. The mandible is especially susceptible to radiation damage due to its dense cortical bone and relatively poor vascularization compared to the maxilla [22].

Alveolar bone and periodontal tissues act as functional units providing tooth support, vascular supply, and mechanical stability. Nevertheless, radiation compromises this balance by inducing collagen cross-linking, reducing cellular turnover, and decreasing osteoblastic activity [23,24]. Consequently, bone demineralization, trabecular rarefaction, and cortical discontinuity can be visualized on diagnostic imaging, most precisely by CBCT, which enables early detection of bone density alterations and microstructural defects [25,26].

In addition, radiation-induced xerostomia resulting from salivary gland hypofunction exacerbates mucosal fragility and increases susceptibility to radiation caries, particularly along smooth and cervical surfaces of the teeth. Also, the reduction in salivary buffering capacity and viscosity alters the oral microbiota, further promoting demineralization [20,25,27,28].

A detailed understanding of tissue-specific radiosensitivity and the underlying cellular and molecular mechanisms is essential for clinicians involved in the care of patients treated with head and neck radiotherapy. Recognizing how mucosa, salivary glands, bone, and periodontal structures respond to radiation, ranging from epithelial apoptosis and cytokine dysregulation to vascular impairment and altered tissue remodeling, provides a necessary biological context for interpreting post-treatment changes and for guiding the safe and appropriate use of dentomaxillofacial imaging in irradiated individuals.

## 4. Tumors of the Oral Cavity and Oropharynx

Radiotherapy remains a cornerstone in the management of head and neck malignancies, particularly for squamous cell carcinomas (SCCs) arising in the oral cavity and oropharynx [29,30]. Together, these neoplasms account for the majority of epithelial tumors in this anatomical region and share overlapping risk factors and treatment paradigms.

### 4.1. Oral Squamous Cell Carcinoma (OSCC)

The oral cavity comprises the lips, tongue, floor of the mouth, oral mucosa, alveolar ridge and hard palate, thus being the site of origin for approximately 90–95% of head and neck SCC [29,31]. These tumors originate from the stratified squamous epithelium, often after chronic exposure to carcinogenic factors such as tobacco and alcohol use, poor oral hygiene, and mechanical irritation [31,32]. Human papillomavirus (HPV) infection plays a lesser role in oral carcinogenesis compared to oropharyngeal sites [31,33,34].

Treatment approaches for OSCC depend on the stage and location of the tumor. Early-stage lesions (T1–T2, N0) are usually managed with surgical excision, followed by adjuvant radiotherapy when high-risk histopathological features are present [35,36,37]. Locally advanced or unresectable tumors are treated with definitive radiotherapy or concurrent chemoradiotherapy, typically delivering doses of 66–70 Gy to the primary tumor and involved nodes [30,35]. Intensity-modulated radiotherapy and image-guided techniques allow precise targeting while minimizing dose to critical oral structures, such as mucosa, salivary glands, and mandible [30,38].

Despite these advances, radiotherapy-induced complications (i.e., mucositis, xerostomia, radiation caries, and osteoradionecrosis) remain significant and require close collaboration between radiation oncologists and dental specialists to optimize oral health and functional outcomes.

### 4.2. Oropharyngeal Squamous Cell Carcinoma (OPSCC)

The oropharynx encompasses the base of the tongue, soft palate, tonsillar region and posterior pharyngeal wall, often being another site of SCC in the head and neck [29]. Unlike OSCC, HPV-related carcinogenesis has emerged as the dominant etiological pathway in OPSCC, particularly in younger, non-smoking individuals [33,34]. These HPV-positive tumors typically overexpress the p16 protein and demonstrate improved prognosis and radiosensitivity compared to their HPV-negative counterparts [39,40].

Therapeutic strategies for OPSCC depend on HPV status, tumor stage, and patient comorbidities [30,39]. For HPV-negative or locally advanced disease, definitive concurrent chemoradiotherapy is the standard treatment, with doses around 70 Gy to the gross primary lesion and involved lymph nodes [30,35,41]. HPV-positive OPSCC, given its favorable biology, is the subject of ongoing de-escalation trials investigating reduced-dose regimens or minimally invasive surgeries followed by adjuvant radiotherapy in order to preserve function while maintaining high cure rates [35,42,43].

From a biological perspective, the proximity of OPSCC lesions to radiosensitive structures, such as base of the tongue, soft palate and salivary glands, demands meticulous dosimetric planning [44,45,46]. Advanced radiotherapy modalities, including chemoradiotherapy and proton therapy, help spare these organs at risk, thus reducing long-term toxicities (e.g., dysphagia, xerostomia, and taste alteration) [35,38,44,45].

In summary, oral and oropharyngeal SCC share a common epithelial origin, but differ aetiologically and biologically. Radiotherapy, either as a primary treatment or as part of a multimodal strategy, continues to play a central role in achieving local control. As modern radiotherapy techniques evolve toward precision and tissue preservation, integration with dental and radiologic care remains critical to mitigate oral complications and ensure long-term functional rehabilitation.

## 5. Impact of Radiation Therapy on the Oral Cavity and Oropharynx

Radiation therapy is one of the primary modalities for treating head and neck cancers, including malignancies of the oral cavity and oropharynx, but its non-selective action on normal tissues leads to significant biological and functional complications. These effects depend on dose, fractionation, and specific structures included in the radiation field [28,29,35,45,47].

In recent years, radiotherapy delivery has advanced considerably, with Intensity-Modulated Radiation Therapy (IMRT) and Volumetric Modulated Arc Therapy (VMAT) providing highly conformal dose distributions and improved sparing of the salivary glands, mandible, and masticatory structures when compared with earlier three-dimensional conformal techniques [38,45]. Image-guided radiotherapy (IGRT) has further enhanced precision by incorporating daily verification and adaptive adjustments in head and neck treatment planning [45]. These developments parallel improvements in imaging technology, including optimized CBCT acquisition protocols, iterative reconstruction algorithms, pulsed exposure systems, and AI-assisted dose modulation, all of which reflect contemporary standards in radiation protection and diagnostic imaging [13,14,48,49,50]. Incorporating these modern techniques into clinical practice is essential for aligning radiotherapeutic precision with biologically informed imaging strategies.

Within the oral cavity, the most radiosensitive structures are the oral mucosa, salivary glands, and alveolar bone [20,23,27,28]. Doses between 20 and 30 Gy trigger damages to epithelial and salivary tissues, resulting in mucositis, the earliest and most common acute toxicity characterized by erythema, pseudomembranous ulceration, pain, and dysphagia [20,27,28]. These lesions arise from epithelial apoptosis and inflammatory cytokine release, and their severity correlates with total dose and concurrent chemotherapy [18,21,28,51].

Salivary gland atrophy leads to xerostomia and hyposalivation at higher doses (i.e., 40–65 Gy), resulting in viscous saliva, fissured lips, and altered taste (dysgeusia). Reduced salivary flow causes radiation caries, which may appear within weeks after therapy, as well as secondary infections, particularly candidiasis and herpetic lesions [19,20,27,28]. These complications are often accompanied by nutritional deficiency and psychological distress due to painful oral function [20].

Chronic and late effects include osteoradionecrosis, usually developing after doses exceeding 60 Gy [19,20,22]. The mandible, because of its dense structure and limited vascularization, is the most affected site. Osteoradionecrosis manifests as necrotic bone exposure, pain, suppuration, and sometimes fistula or pathological fracture [22,23]. Risk factors include poor oral hygiene, tobacco and alcohol use, and dental extractions after radiation therapy [19,20,22]. Additionally, trismus (resulting from fibrosis of masticatory muscles) commonly appears three to six weeks post-treatment, thus limiting oral hygiene and nutrition [52,53].

It is important to note that, to date, no clinical evidence indicates that dentomaxillofacial radiology (whether intraoral radiographs, panoramic imaging, or CBCT) induces or increases the risk of osteoradionecrosis in previously irradiated bone. The development of osteoradionecrosis remains primarily associated with high-dose therapeutic irradiation, local trauma, infection, and compromised vascularity, rather than with the very low additional doses delivered by diagnostic imaging [19,22,26,28]. On the contrary, imaging plays an essential role in the diagnosis, staging, and monitoring of osteoradionecrosis and is indispensable for planning preventive and therapeutic interventions in affected patients.

In the oropharynx, radiation causes inflammation and fibrosis in the base of the tongue, soft palate and tonsillar region, leading to dysphagia, dysgeusia, and speech alterations [2,20,28,30,47]. Opportunistic infections may also arise due to immune suppression [2]. Altogether, these complications highlight the profound impact of radiotherapy on oral and oropharyngeal tissues, which emphasizes the need for early dental evaluation, continuous oral care, and close interdisciplinary management to prevent and control radiation-induced toxicity [2,17,19,28].

### Cellular and Molecular Impact of Radiation on the Oral Mucosa

**Figure 2:**
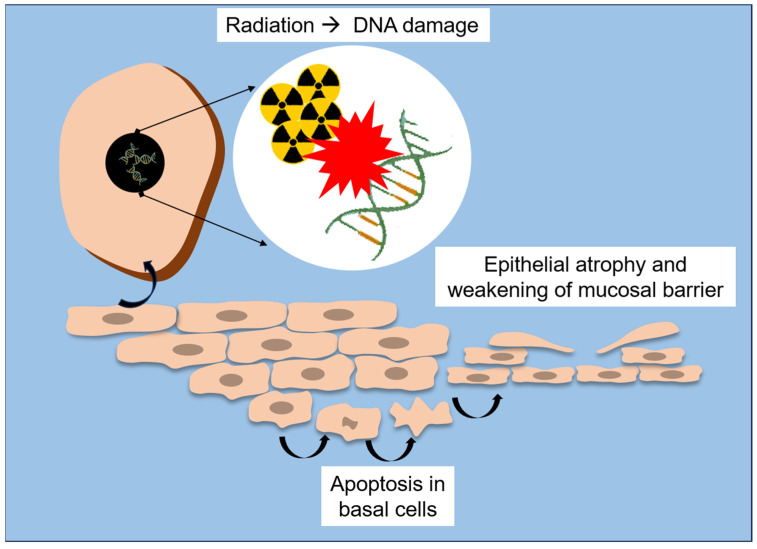
Schematic representation of radiation-induced structural and molecular lesions to the oral mucosa.

Radiation therapy exerts profound structural and molecular alterations on the oral mucosa, compromising its integrity and regenerative capacity. At the tissue level, ionizing radiation causes epithelial atrophy, vascular damage and inflammation, with early manifestations (e.g., erythema and edema) often progressing to mucositis, ulceration, and secondary infections [18,20,25]. This reduction in proliferative activity precedes visible mucosal lesions, highlighting that cellular depletion occurs before clinical signs of mucositis appear [18,51]. A schematic overview of these early structural and molecular events is presented in Figure 2.

At the ultra-structural level, Asikainen et al. [25] revealed that radiation leads to disruption of epithelial microplicae (MPL), which are membrane ridges supporting the salivary pellicle and acting as a barrier against microorganisms. By using scanning and transmission electron microscopy, they found partial or total loss of MPL and desmosomal junctions, accompanied by vesiculation of epithelial cells and thickening of intercellular spaces, particularly in patients who developed osteoradionecrosis. These findings suggest that destruction of the microarchitecture of mucosal surface directly weakens the epithelial barrier, which may contribute to microbial invasion and impair healing in irradiated bone and soft tissue.

At the molecular level, studies of radiation-induced oral mucositis demonstrate a complex interplay between inflammatory signaling, oxidative stress, and impaired repair pathways. According to Saul-McBeth et al. [18], radiation activates the NF-κB, IL-1, and IL-17 signaling cascades, which amplifies the release of pro-inflammatory cytokines (i.e., IL-6, TNF-α) and matrix metalloproteinases (i.e., MMP-3, MMP-9, MMP-10), thus degrading the extracellular matrix and delaying mucosal repair. In murine models, deficiency of IL-17 receptor (IL-17RA) markedly aggravated oral mucositis by resulting in deeper ulcerations, increased neutrophil infiltration, and higher oxidative stress. Conversely, intact IL-17 signaling was shown to protect mucosal integrity, thus promoting epithelial proliferation (Ki-67 expression), expression of keratinocyte growth factor (KGF), and secretion of tissue repair mediators (e.g., IL-24 and MMP-3).

These cellular and molecular mechanisms explain the continuum from acute mucosal inflammation to chronic tissue injury. Initially, DNA damage and accumulation of reactive oxygen species (ROS) lead to apoptosis of basal epithelial cells [18,25]. Subsequent loss of mucosal barrier function exposes the underlying connective tissue, which triggers an inflammatory cascade perpetuating pain, ulceration, and infection [54]. Without adequate epithelial regeneration or immune balance, chronic hypoxia and fibrovascular impairment contribute to late complications such as fibrosis and osteoradionecrosis [22,23,47].

Altogether, the evidence from histological, ultra-structural, and molecular studies underscores that radiation-induced injuries in the oral cavity is not merely a surface phenomenon, but reflects profound disruption of epithelial homeostasis, immune signaling, and barrier function. Understanding these mechanisms provides a biological basis for developing targeted therapies, such as anti-inflammatory cytokine modulators, antioxidant treatments, and epithelial growth factor analogues to mitigate mucosal toxicity and enhance post-radiation healing in head and neck cancer patients.

Importantly, radiographic and clinical examinations should not be omitted after head and neck cancer treatment. International guidelines emphasize that early detection of recurrence, metastasis, and treatment-related complications depends on frequent follow-up visits. The American Head and Neck Society recommends examinations every 1 to 3 months during the first year after radiotherapy, as most recurrences occur within this period and delayed diagnosis significantly worsens prognosis [55]. In addition to oncologic surveillance, dentomaxillofacial imaging plays an essential role in dental management of irradiated patients. Radiotherapy significantly increases the risk of radiation caries, hyposalivation, pulp sensitivity alterations, apical periodontitis, and osteomyelitis of the jaw, making radiographic assessment indispensable for determining lesion depth, pulpal proximity, and periapical status [24,26,28,55,56]. In complex endodontic cases, CBCT provides superior three-dimensional visualization and reduces the likelihood of untreated canals, perforations, or inadequate debridement, which are known risk factors for osteoradionecrosis and post-radiotherapy infections. Therefore, imaging examinations remain a key element for safe and effective dental care in this vulnerable population and should be performed with optimized protocols but never withheld when clinically indicated.

## 6. Potential Additive Mechanisms of Low-Dose Dentomaxillofacial Imaging in Patients Undergoing Head and Neck Radiotherapy

Although dentomaxillofacial diagnostic imaging delivers radiation doses several orders of magnitude lower than those used in therapeutic radiotherapy, its biological effects warrant careful consideration in patients currently undergoing or recently completing head and neck radiotherapy. Overlap of anatomical regions and altered micro-environment of irradiated tissues can result in additive or synergistic effects, even when individual imaging doses are minimal [57].

### 6.1. Comparative Radiation Doses

Dental radiographic procedures, including intraoral, panoramic, and CBCT scans, deliver very low doses of ionizing radiation when considered individually. However, their timing relative to head and neck radiotherapy and the cumulative exposure of radiosensitive oral tissues, particularly mucosa, salivary glands and mandible, may influence local biological responses [11,35,48,49,50], as detailed in Table 1.

Although the therapeutic dose of radiotherapy (60–70 Gy) far exceeds that of any diagnostic exposure, dentomaxillofacial imaging targets the same critical structures already affected by therapeutic irradiation [15,20,28,35]. Consequently, repeated low-dose exposures during or shortly after radiotherapy may biologically amplify ongoing or latent radiation-induced injuries through additive oxidative or inflammatory mechanisms [18,47].

To make the dose contrast more explicit, Table 2 summarizes estimated organ doses delivered during common dentomaxillofacial imaging procedures relative to typical therapeutic exposures received by the same tissues during head and neck radiotherapy [35,48].

While low-dose dentomaxillofacial imaging raises theoretical concerns regarding additional oxidative or inflammatory stress in previously irradiated tissues, it is essential to emphasize that such examinations are indispensable for comprehensive post-radiotherapy dental care. Radiation therapy markedly increases the incidence of radiation caries, which often progresses rapidly and requires radiographic assessment to determine the extent of dentin involvement and proximity to the pulp chamber [24,56]. Without adequate imaging, pulpitis and endodontic complications may be overlooked. CBCT is also critical in complex root canal anatomies, helping prevent persistent infection, periapical lesions, and treatment failure, conditions that are known risk factors for osteomyelitis and osteoradionecrosis in irradiated bone [26,58]. Thus, imaging should not be avoided; instead, the focus should be on selecting the smallest field of view (FOV) and the lowest dose compatible with diagnostic accuracy.

### 6.2. Biological Basis for Additive Effects

Experimental and histological studies have shown that ionizing radiation, even at diagnostic levels, can trigger oxidative stress, DNA strand breaks, and apoptotic signaling in epithelial cells [10,59]. In healthy patients, these effects are transient and repaired by intact DNA repair pathways. However, in irradiated tissues, where vascularization, cellular turnover, and immune regulation are already impaired, such exposures may exceed the diminished repair capacity [60,61,62].

After radiotherapy, the oral mucosa exhibits persistent inflammation and altered cytokine expression (i.e., IL-1, IL-6, TNF-α). Re-exposure, even to low diagnostic doses, could re-activate these inflammatory cascades, promoting subclinical cytotoxicity, delayed epithelial recovery, and heightened susceptibility to mucositis or secondary infections. Similarly, the salivary gland tissue, which is already compromised by fibrosis and acinar atrophy, may be affected by transient functional stress under additional exposure, thus further aggravating xerostomia and altered salivary composition [18].

The biological recovery of irradiated oral tissues follows heterogeneous and often prolonged timelines. Epithelial turnover, which normally occurs every 7 to 14 days, may require several weeks to return to baseline after radiotherapy because basal keratinocyte compartments remain depleted and cytokine signaling remains dysregulated during the subacute phase [63]. Vascular regeneration is even slower: endothelial injury, fibrosis, and microvascular rarefaction may persist for months or years, contributing to sustained hypoxia and impaired tissue remodeling [62,64]. Similarly, salivary glands demonstrate limited regenerative potential, with acinar atrophy and ductal dysfunction often remaining long after treatment. Bone healing is particularly delayed due to reduced osteoblastic activity, altered collagen deposition, and chronic fibroatrophic changes [27,28,56]. These prolonged or incomplete recovery processes help explain why irradiated tissues may remain biologically fragile even when the physical dose from diagnostic imaging is minimal, reinforcing the need for careful timing and adherence to radiological protection principles [57,64].

### 6.3. Dosimetric and Tissue-Specific Considerations

Dosimetric analyses reveal that salivary glands, oral mucosa, and thyroids are among the most irradiated organs during dentomaxillofacial imaging, with CBCT showing the widest dose variability. Depending on the FOV and acquisition parameters, glandular doses may increase 10–20-fold compared to panoramic radiographs. These same tissues, when previously irradiated to 40–60 Gy during radiotherapy, remain biologically fragile for months. Hence, CBCT performed during or shortly after radiotherapy could deliver small but spaced coincident doses to tissues with reduced vascularity, leading to an “amplified biological effect” despite a negligible physical dose contribution [25].

### 6.4. Timing and Cumulative Biological Stress

The likelihood of additive effects depends strongly on the temporal relationship between diagnostic imaging and course of the radiotherapy. During the acute phase (weeks 2–6), the mucosa’s proliferative compartment is highly depleted, and any added oxidative insult may delay regeneration [63]. Later, in the chronic phase, when fibrosis and hypoxia dominate, even minor additional exposures could promote endothelial dysfunction and low-grade inflammatory responses, thus contributing to long-term tissue remodeling [57,64].

While modern radiotherapy modalities such as IMRT and proton therapy substantially reduce the dose delivered to salivary glands, mandible, and other organs at risk when compared with conventional Three-Dimensional Conformal Radiation Therapy (3D-CRT) [44,45], irradiated tissues may still exhibit chronic hypovascularity, fibrosis, and impaired remodeling. These biological alterations persist regardless of the delivery technique, and there is currently no evidence to suggest that dentomaxillofacial imaging recommendations should differ according to the radiotherapy modality a patient has received. Therefore, the same principles of justification and dose optimization apply to all previously irradiated individuals.

### 6.5. Clinical Implications and Radioprotective Practices

To minimize potential additive effects of diagnostic imaging during radiotherapy, key clinical considerations and radioprotective practices are summarized in Table 3 [11,49,50]:

The radiation dose delivered by CBCT is strongly influenced by several acquisition parameters. The FOV is the primary determinant of patient exposure, with larger volumes irradiating a greater amount of tissue and significantly increasing glandular and osseous doses. Tube voltage (kVp) and tube current (mA) also play a critical role: higher kVp increases the penetrating power of the beam and raises the absorbed dose, whereas higher mA increases photon quantity and proportionally elevates dose. Exposure time and the use of continuous versus pulsed emission further modify total output. Voxel size is another relevant factor, as smaller voxels require higher exposure settings to maintain image noise within diagnostically acceptable limits. Therefore, reducing FOV, lowering mA/kVp when compatible with diagnostic requirements, using pulsed exposure, and selecting larger voxel sizes in non-critical evaluations represent practical strategies to minimize dose while maintaining adequate image quality [13,48,49,50].

Beyond these technical adjustments, clinical decision-making should be individualized and multidisciplinary. Radiation oncologists, medical physicists, and dentomaxillofacial radiologists must jointly evaluate whether additional CBCT or other imaging will meaningfully alter patient management. For example, by detecting significant anatomical changes, verifying target coverage, or identifying early complications that could modify the treatment plan. In borderline cases, alternative strategies such as careful clinical examination, or re-using previously acquired data should be considered before exposing patients to further ionizing radiation.

Despite the fact that the use of small FOV is recommended for all patients undergoing CBCT, this principle becomes especially important in individuals previously treated with head and neck radiotherapy, given their reduced vascularity, impaired tissue repair, and increased susceptibility to chronic inflammation. Nonetheless, the rationale for FOV minimization follows general radiological protection principles and is not exclusive to this population [13,48].

**Figure 3:**
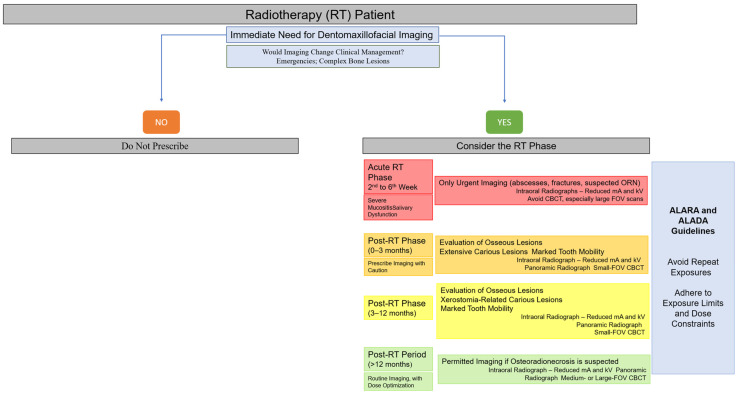
Clinical decision-making flowchart for dentomaxillofacial imaging in head and neck radiotherapy patients.

To facilitate clinical decision-making, a visual flowchart summarizing when imaging should or should not be performed during the different phases of radiotherapy has been included (Figure 3). This flowchart integrates the principles of justification, timing, and dose optimization described in this section, providing clinicians with a practical tool for safe imaging selection in irradiated patients [2,13,21,22,26,28,35,47,48,49,50,55,63,64].

In addition to radiobiological and clinical considerations, dose optimization in dentomaxillofacial imaging also aligns with broader sustainability principles in healthcare. Sustainable Development Goal 3 (Good Health and Well-Being) emphasizes safe, equitable, and ethically responsible use of medical technologies. Recent discussions in nuclear medicine and radiopharmaceutical sciences have highlighted how optimizing radiation exposure, reducing unnecessary imaging, and integrating resource-efficient practices contribute to sustainable healthcare delivery [65,66,67]. Though these works focus on nuclear medicine, the underlying concepts directly support the rationale for adopting justification-based, biologically informed, and protocol-optimized imaging strategies in dentomaxillofacial radiology, particularly for patients previously exposed to therapeutic head and neck irradiation.

## 7. Conclusions

Even though diagnostic dentomaxillofacial imaging contributes to only a small fraction of the total radiation dose received by patients with head and neck cancer, its biological impact on previously irradiated oral tissues should not be neglected. Even low-dose X-rays can act as secondary biological stressors, thus being capable of re-activating inflammatory, oxidative, and apoptotic pathways within mucosa, salivary glands and alveolar bone, all tissues already compromised by therapeutic exposure [25,68].

In this context, the principles of justification and optimization, known, respectively, as “As Low As Reasonably Achievable” (ALARA) and “As Low As Diagnostically Acceptable” (ALADA), must guide every diagnostic decision [48,49]. Imaging should be performed only when the expected clinical benefit outweighs the potential biological risk by using the lowest dose and smallest FOV for diagnostically adequate results [48]. Special attention should be paid to the examination time by avoiding periods of acute mucositis or active inflammation and to the selection of imaging modalities favoring small-field CBCT or low-dose digital radiography when appropriate [48,49,50].

Furthermore, a comprehensive radioprotective approach should extend beyond simple dose reduction. Incorporating biological awareness, interdisciplinary planning, and post-exposure supportive measures (e.g., antioxidant therapy, saliva substitutes, and strict oral hygiene) can significantly improve tissue resilience and healing [20,69,70,71]. The integration of modern low-dose imaging technologies and artificial intelligence-based exposure optimization systems represents a promising possibility for personalized radioprotection [72,73,74,75], thus allowing clinicians to modify imaging parameters according to the patient’s prior radiation history and tissue vulnerability. Practical strategies supported by clinical evidence include the use of antioxidant agents such as vitamin E (400–800 IU/day) and pentoxifylline (400 mg, three times daily), which have demonstrated benefits in reducing fibroatrophic changes and improving post-radiotherapy tissue recovery [69,70]. Saliva substitutes formulated with carboxymethylcellulose or xanthan gum have also been shown to improve lubrication, swallowing ability, and nutritional status in irradiated patients [71]. Incorporating these supportive therapies into routine care may enhance mucosal protection and functional rehabilitation, complementing radiological dose optimization strategies.

In conclusion, the safe use of diagnostic imaging in patients undergoing or recovering from head and neck radiotherapy depends on a synergistic balance between diagnostic necessity and radiobiological caution.

## Figures and Tables

**Table 1:** Comparative radiation doses in dentomaxillofacial radiology and therapeutic head and neck radiotherapy.

Radiation Level	Modality	Typical Effective Dose (µSv)	Equivalent to Background (Bk)
**Very Low** 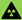	Intraoral (periapical/bitewing)	0.3–1.4	<1 day Bk
**Low** 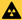 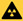	Bitewing (4 images)	3.4–5.0	<1 day Bk
	Cephalometric	2–10	<1–1.3 days Bk
	Panoramic (CCD)	14–30	≈3 days Bk
**Moderate** 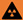 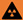 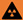	Panoramic (PSP)	19–75	≈2–9 days Bk
	CBCT (small FOV, pediatric) CBCT (small FOV, adult)	7–521 19–652	≈1–61 days Bk ≈2–77 days Bk
	CBCT (medium FOV, adult)	45–360	≈5–101 days Bk
**High** 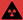 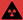 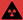 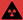	CBCT (large FOV, adult)	68–1073	≈8–126 days Bk
	Fan-beam CT (Head and neck)	860–1500	≈3–6 months Bk
	Head and neck radiotherapy course	≈70 Gy = 7 × 10^7^ µSv	≈70,000,000 µSv (≈10^6^ × diagnostic dose)

Note: Values for dentomaxillofacial imaging adapted from Benavides et al. [48]. The therapeutic dose for head and neck radiotherapy (≈70 Gy = 7 × 10^7^ µSv) is included for comparison only; effective dose (µSv) is not formally applicable to radiotherapy [9,35]. CCD = charge-coupled device; PSP = phosphor storage plate; FOV = field of view. Color shading in the left column indicates the radiation-dose category assigned to each modality: green denotes very low dose, yellow indicates low dose, orange represents moderate dose, and red identifies high-dose examinations.

**Table 2:** Estimated dose to key head and neck tissues during common dentomaxillofacial radiology compared with therapeutic radiation exposure.

Tissue/Organ	Typical Dose from Dental Imaging	Typical Dose from H&N Radiotherapy	Relative Magnitude
Oral mucosa	Intraoral radiograph: 0.5–1.0 mGy Panoramic: 1–5 mGy CBCT (small FOV): 5–20 mGy	50–70 Gy	~10^4^–10^6^× higher in RT
Salivary glands (parotid/submandibular)	Panoramic: 1–3 mGy CBCT: 5–30 mGy	30–60 Gy	~10^4^–10^5^× higher in RT
Mandible/Alveolar bone	Intraoral: 1–3 mGy CBCT small FOV: 10–100 mGy	50–70 Gy	~10^3^–10^5^× higher in RT
Thyroid	Panoramic: 0.5–1.0 mGy CBCT: 1–30 mGy	<5 Gy scattered dose (depending on field)	~10^2^–10^3^× higher in RT

Note: Values derived from published dosimetry studies of dental radiography and CBCT. Therapeutic dose estimates correspond to standard head and neck radiotherapy protocols delivering 60–70 Gy to mucosa, mandible, and salivary glands. (RT: Radiotherapy).

**Table 3:** Radioprotective strategies for dentomaxillofacial radiology to minimize additive exposure during head and neck radiotherapy.

Aspect	Description
**Clinical justification**	Each imaging examination should be strictly necessary and clinically indispensable during radiotherapy.
**Protocol optimization**	Use small FOV, pulsed exposure, and reduced mA/kVp CBCT settings whenever compatible with diagnostic quality.
**Appropriate timing**	Schedule imaging outside periods of acute mucositis or heightened inflammatory activity, when tissues are more vulnerable to injury.
**Protection of critical organs**	Employ thyroid collars, precise collimation, and avoid redundant or unnecessary acquisitions.
**Cumulative dose monitoring**	Record and track all imaging exposures within the oncology record to monitor the patient’s total radiation burden.

## Data Availability

No new data were created or analyzed in this study. Data sharing is not applicable to this article.
